# The role and mechanisms of action of microRNAs in cancer drug resistance

**DOI:** 10.1186/s13148-018-0587-8

**Published:** 2019-02-11

**Authors:** Wengong Si, Jiaying Shen, Huilin Zheng, Weimin Fan

**Affiliations:** 1 Program of Innovative Cancer Therapeutics, Division of Hepatobiliary and Pancreatic Surgery, Department of Surgery, First Affiliated Hospital, College of Medicine, Zhejiang University, 79 Qingchun Road, Hangzhou, 310003 Zhejiang Province China; 2 Key Laboratory of Organ Transplantation, Hangzhou, 310003 Zhejiang Province China; 3 Key Laboratory of Combined Multi-organ Transplantation, Ministry of Public Health, Hangzhou, 310000 China; 4 Department of Medical Oncology, Sir Run Run Shaw Hospital, College of Medicine, Zhejiang University, Hangzhou, Zhejiang China; 5 Clinical Research Center, First Affiliated Hospital of Zhejiang University College of Medicine, Hangzhou, 310000 China; 6 Department of Pathology and Laboratory Medicine, Medical University of South Carolina, Charleston, SC 29425 USA

**Keywords:** MicroRNAs, Drug resistance, Cancer, Dysregulation, Mechanisms, Chemotherapy

## Abstract

MicroRNAs (miRNAs) are small non-coding RNAs with a length of about 19–25 nt, which can regulate various target genes and are thus involved in the regulation of a variety of biological and pathological processes, including the formation and development of cancer. Drug resistance in cancer chemotherapy is one of the main obstacles to curing this malignant disease. Statistical data indicate that over 90% of the mortality of patients with cancer is related to drug resistance. Drug resistance of cancer chemotherapy can be caused by many mechanisms, such as decreased antitumor drug uptake, modified drug targets, altered cell cycle checkpoints, or increased DNA damage repair, among others. In recent years, many studies have shown that miRNAs are involved in the drug resistance of tumor cells by targeting drug-resistance-related genes or influencing genes related to cell proliferation, cell cycle, and apoptosis. A single miRNA often targets a number of genes, and its regulatory effect is tissue-specific. In this review, we emphasize the miRNAs that are involved in the regulation of drug resistance among different cancers and probe the mechanisms of the deregulated expression of miRNAs. The molecular targets of miRNAs and their underlying signaling pathways are also explored comprehensively. A holistic understanding of the functions of miRNAs in drug resistance will help us develop better strategies to regulate them efficiently and will finally pave the way toward better translation of miRNAs into clinics, developing them into a promising approach in cancer therapy.

## Background

Cancer is a serious threat to human life and health, and in recent years, it has become a leading cause of death in humans. According to statistical reports, new cases of cancer reached 14.1 million worldwide, and the total number of cancer-related deaths reached 8.2 million in 2012. With an increase in life expectancy and deterioration of the global ecosystem, the incidence of cancer is increasing. It is expected that the number of new cases will reach 23.6 million by 2030 [1].

Currently, chemotherapy, radiotherapy, and surgery are the most common cancer therapies. For cancers such as lymphoma, leukemia, small cell lung cancer, chemotherapy is the first line of treatment. For other solid tumors, chemotherapy can be used as an auxiliary treatment to eliminate postoperative residual nodules to prevent relapse or as pre-local tumor before surgery or radiotherapy. In addition, chemotherapy is also used as palliative care in patients who cannot undergo radical surgery [2]. In recent decades, chemotherapy drugs have made great progress, but the occurrence of tumor drug resistance often leads to treatment failure. For advanced cancer patients, drug-resistance is a major obstacle to successful treatment [3]. According to statistical reports, more than 90% of deaths of tumor patients are associated with chemotherapeutic drug resistance [4, 5]. Overall, drug resistance can be divided into endogenous and acquired drug resistance, and the underlying mechanisms need to be elucidated. At present, it is believed that the increase in drug efflux, target switch, cell cycle checkpoints alteration, apoptosis inhibition, and increase in DNA damage repair are all related to drug resistance [6].

**Fig. 1:**
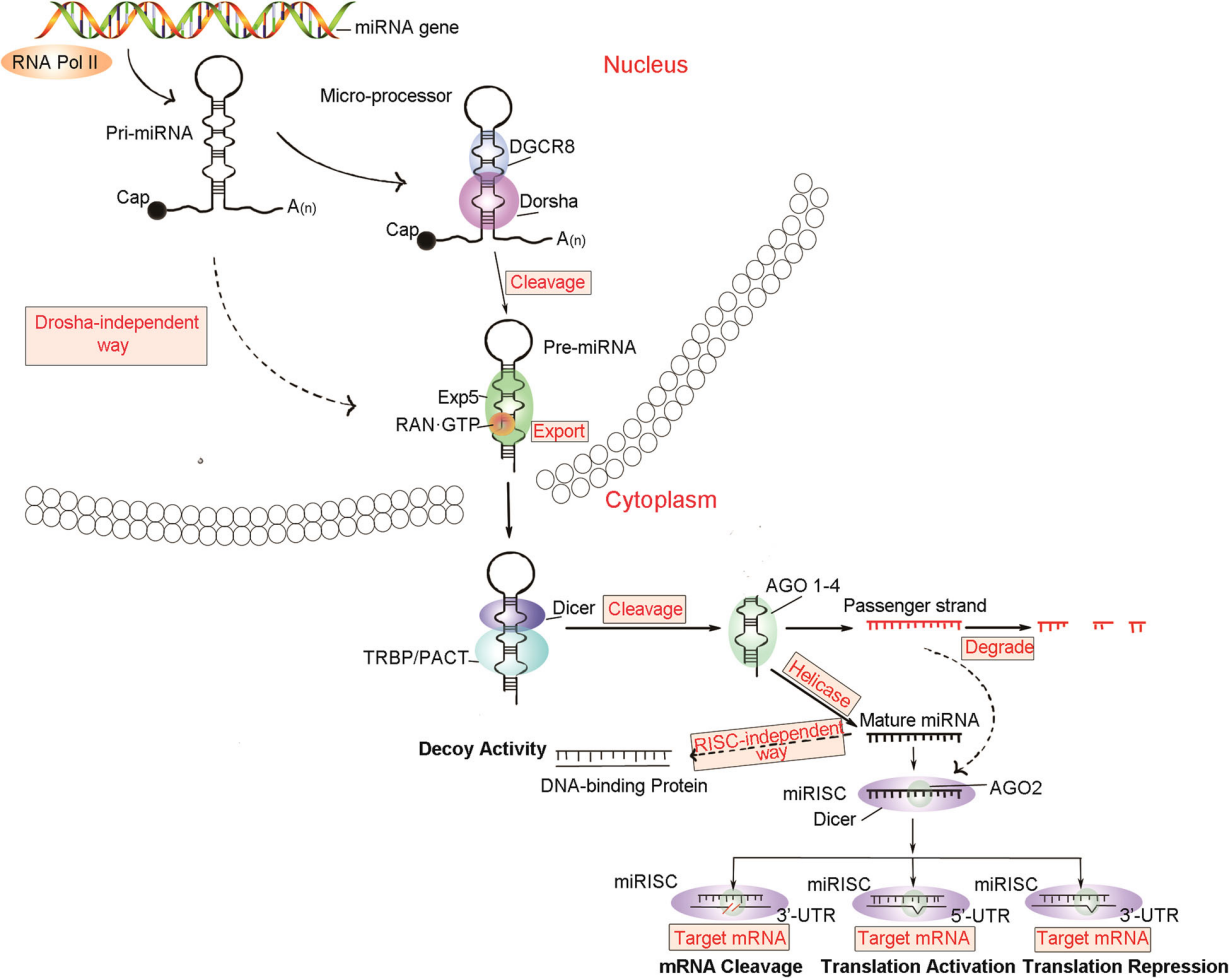
The mechanisms of microRNA biogenesis and its regulation of gene expression. The solid arrows represents the classical pathway, the dotted arrows represents the non-classical pathway

MiRNAs are small non-coding RNAs with a length of approximately 19–25 nt, which can regulate various target genes. MiRNAs are involved in the regulation of a variety of biological processes, such as cell cycle, differentiation, proliferation, apoptosis, stress tolerance, energy metabolism, and immune response [7]. The biogenesis of miRNAs in animal cells and the mechanisms of regulation of their target gene expression are shown in Fig. 1. In simple terms, this process can be divided into the following steps [8, 9]: (1) the miRNA gene is transcribed into primary miRNA (pri-miRNA) by RNA polymeraseII(RNA polII) in the nucleus; (2) pri-miRNA is processed by the Drosha/DGCR8 complex to release the intermediate precursor miRNA (pre-miRNA), which is approximately 70 nt with a stem loop structure and a 2 nt overhang at the 3′-end; (3) pre-miRNA binds to the Exp5/Ran-GTP complex, which allows for its transport into the cytoplasm; (4) the pre-miRNA is then processed into double-stranded RNA by the Dicer/TRBP/PACT complex in the cytoplasm; (5) the miRNA-duplex is unwound into single strands by the action of helicase. Under normal circumstances, the RNA strand with lower stability at the 5′-end will be integrated into the RNA-induced silencing complex (RISC) and become a mature miRNA, and the strand with higher stability at the 5′-end will be degraded; (6) miRNA-induced silencing complex (miRISC) will bind to the 3′-untranslated regions (UTR) of the target mRNA, thus inhibiting its translation.

In plant cells, miRISC will degrade its target mRNA, and the biogenesis of miRNAs is slightly different from that in animal cells [10].

Existing research shows that this classical processing and functioning pathway has some exceptions. For example, in step (2) of its biosynthesis process, the pri-miRNA can also be processed into pre-miRNA in a Drosha-independent way [11]. In step (5), the two strands may be randomly integrated into RISC, or they could bind to mRNA in RISC-independent manner [12]. In step (6), some miRISC can bind to the 5′-UTR of mRNA, upregulating its translation [13, 14].

Through the above approach, miRNAs regulate about 30% of human genes [15]; half of these genes are tumor-associated or located in fragile loci [16]. The deregulation of miRNAs in tumor cells suggests that they have modulatory effects on tumor development [17]. In fact, some miRNAs may act as tumor genes, and others can act as tumor suppressor genes. Interestingly, some can act as both, depending on the tissue where they are being expressed [7, 18–20]. In recent years, a large number of studies have indicated that many of the genes regulated by miRNAs are related to the response of tumor cells to chemotherapeutic agents [21]. According to miRNA expression profiles in different tumor cells, which were characterized by using microarray, miRNA sequencing or high-throughput screening technology, a lot of miRNAs were found to be involved in the regulation of cellular response to drugs. This article will review the role of miRNAs in drug resistance, and their targets in different tumor cells. Next, due to the frequent changes in miRNA expression profiles in drug-resistant tumor cells, we will analyze the mechanisms of miRNA dysfunction. Finally, we will focus on the mechanisms of miRNAs in regulating tumor drug resistance.

## The role of miRNAs in drug-resistance regulation in different types of cancer

Through deep sequencing, high-throughput screening, and chip technologies, it has been found that there are many deregulated miRNAs in cancer cells. These miRNAs regulate different target genes, including genes that affect the response of cells to chemotherapy drugs. In Table 1, we list some of the reported miRNAs that regulate drug resistance and have clear targets in common cancers. Because of the tissue specificity of miRNA regulation [19, 20], one kind of miRNA can target multiple mRNAs, and one mRNA can also be targeted by multiple miRNAs [22, 23]; therefore, the same miRNA may play different, even opposite, roles in regulating drug resistance in different cancer cells.

**Table 1:** MiRNAs involved in drug resistance regulation in various cancers

MiRNA	Cancer type	Corresponding drug	Targets	References
- Let-7b	Breast	−/TAM	*ER-α36*	[35]
	Ovarian	+/Taxanes	*IMP-1*	[82]
- Let-7c	NSCLC	−/CDDP	*ABCC2, BCL-XL*	[57]
- Let-7e	Ovarian	−/CDDP	*EZH2, CCND1*	[194]
- 9	Ovarian	−/CDDP	*BRCA1*	[83]
- 10b	Colorectal	+/5-FU	*BIM*	[73]
- 15a/16	Breast	−/TAM	*BCL-2 (BCL2)*	[167]
- 15b	Lung	+/CDDP	*PEBP4*	[64]
- 17-5p	Lung	−/PTX	*Beclin1*	[195]
	MCL	+/Topotecan	*PHIPP2*	[196]
	Breast	−/Taxol	*NCOA3*	[197]
- 20a	Colorectal	+/5-FU,L-OHP, Teniposide	*BNIP2*	[74]
	Breast	−/mutidrug	*MAPK1*	[198]
- 21	Ovarian	+/CDDP	*PDCD4*	[88]
	Gastric	+/Trastuzumab	*PTEN*	[108]
	Pancreas	+/GEM	*FasL*	[172]
	Colorectal	+/5-FU	*hMSH2*	[72]
- 23a	Colorectal	+/5-FU	*APAF-1*	[75]
- 25	Gastric	+/CDDP	*FOXO3a*	[199]
- 26a/b	HCC	+/DOX	*ULK1*	[200]
- 26b	HCC	−/DOX	*TAK1, TAB3*	[201]
- 27a	Bladder	+/CDDP	*SLC7A11*	[202]
	Lung	+/CDDP	*RKIP*	[65]
- 30a	Cervical,Breast, HCC	−/CDDP	*Beclin1*	[112]
- 30c	Breast	−/DOX	*YWHAZ*	[203]
- 30e	CML	−/Imatinib	*ABL*	[103]
- 31	NSCLC	+/CDDP	*ABCB9*	[58]
	Ovarian	−/PTX	*MET*	[98]
- 34a	Breast	−/DOX	*NOTCH1*	[204]
	Lung	−/CDDP	*PEBP4*	[67]
	Breast	+/Docetaxel	*BCL-2, CCND1*	[37]
	Prostate	−/PTX	*SIRT1, BCL-2*	[102]
	Colorectal	−/5-FU	*c-Kit*	[77]
- 34c	Lung	+/PTX	*BMF*	[205]
- 92b	NSCLC	+/CDDP	*PTEN*	[68]
- 93	Ovarian	+/CDDP	*PTEN*	[89]
- 96	Colorectal	−/5-FU	*XIAP*	[76]
	Breast, HCC	−/CDDP	*RAD51, REV1*	[43]
- 100	Pancreatic	−/CDDP	*FGFR3*	[114]
	Lung	−/Docetaxel	*PIK1*	[206]
- 101	Ovarian	+/DOX	*EZH2*	[207]
- 103	Leukemia	−/CDDP	*COP1*	[208]
- 106a	Lung	+/CDDP	*ABCA1*	[63]
	Ovarian	+/CDDP	*PDCD4*	[90]
	Ovarian	−/CDDP	*Mcl-1*	[92]
- 122	Colon	−/5-FU	*PKM2*	[209]
- 125b	HCC	−/5-FU	*Hexokinase II*	[38]
	Breast	+/5-FU	*E2F3*	[210]
	APL	+/DOX	*Bak1*	[107]
- 126	NSCLC	−/DOX, VCR	*VEGFA*	[175]
- 130a	Ovarian	−/CDDP	*XIAP*	[93]
	HCC	+/CDDP	*RUNX3*	[46]
	Ovarian	+/CDDP	*PTEN*	[91]
- 130b	Ovarian	−/CDDP, PTX	*CSF-1*	[85]
	Breast	+/DOX	*PTEN*	[211]
- 133a	HCC	−/DOX	*ABCC1*	[52]
- 134	Lung	+/Gefitinib	*MAGI2*	[212]
- 135a/b	Lung	−/CDDP	*Mcl-1*	[171]
- 136	Ovarian	−/PTX	*Notch3*	[213]
- 137	Breast	−/DOX	*YB-1*	[27]
	Multiple myeloma	−/Bortezomib, Eprirubicin	*AURKA*	[214]
- 138	NSCLC	−/CDDP	*ERCC1*	[59]
	NSCLC	−/Gefitinib	*GPR124*	[69]
- 141	Esophageal	+/CDDP	*YAP1*	[215]
	HCC	+/5-FU	*Keap1*	[53]
- 143	Prostate	−/Docetaxel	*KRAS*	[101]
- 145	Ovarian	−/PTX	*SP1, CDK6*	[216]
- 148a	Prostate	−/PTX	*MSK1*	[100]
- 149	Breast	−/DOX	*NDST1*	[28]
- 153	Colorectal	+/L-OHP, CDDP	*FOXO3a*	[78]
- 155	Breast	+/DOX, VP-16, PTX	*FOXO3a*	[36]
- 181a	Breast	−/DOX	*BCL-2*	[32]
	Cervical	+/CDDP	*PRKCD*	[217]
- 181b	Lung	−/VCR, CDDP	*BCL-2*	[109]
- 182	NSCLC	+/CDDP	*PDCD4*	[60]
	HCC	+/CDDP	*TP53INP1*	[44]
- 183	Nasopharyngeal	−/CDDP	*MTA1*	[218]
- 190b	HCC	+/Insulin	*IGF-1*	[219]
- 193a-3p	HCC	+/5-FU	*SRSF2*	[54]
- 193b	HCC	−/Sorafenib	*Mcl-1*	[48]
- 195	Breast	−/DOX	*Raf-1*	[29]
	Colon	−/DOX	*BCL2L2*	[165]
	HCC	+/5-FU	*BCL-w*	[55]
	Colon	−/5-FU	*CHK1, WEE1*	[220]
- 197	Ovarian	+/Taxol	*NLK*	[221]
- 199a-3p	HCC	−/DOX	*mTOR, c-Met*	[173]
- 199a-5p	HCC	−/CDDP	*ATG7*	[47]
- 199b-5p	Ovarian	−/CDDP	*JAG1*	[86]
- 200b	Prostate	−/Docetaxel	*Bmi1*	[222]
	SCLC	−/CDDP,DOX,VP-16	*ZEB2*	[71]
	Lung	−/Docetaxel	*E2F3*	[70]
	Gastric, Lung	−/VCR, CDDP	*BCL-2, XIAP*	[169]
- 200c	Gastric	−/CDDP	*RhoE*	[110]
	Breast	−/DOX	*TrKB, Bmi1*	[223]
	Ovarian	−/PTX	*TuBB3, TrKB*	[96]
	Breast	−/Trastuzumab	*ZNF217, ZEB1*	[224]
- 203	Glioblastoma	−/Imatinib,VP-16,TMZ	*SNAI2*	[225]
	Colorectal	+/L-OHP	*ATM*	[226]
	Colorectal	−/5-FU	*TYMS*	[79]
	Breast	+/CDDP	*SOCS3*	[39]
- 205	NSCLC	+/CDDP	*PTEN*	[61]
- 214	Cervical	−/CDDP	*Bcl2l2*	[111]
	Ovarian	+/CDDP	*PTEN*	[156]
- 216a/217	HCC	+/Sorafenib	*PTEN, SMAD7*	[49]
- 218	Breast	−/GEM	*BIRC5*	[227]
	Gallbladder	−/DOX, Taxol	*PRKCE*	[228]
- 221	Breast	+/Trastuzumab	*PTEN*	[229]
- 223	HCC	−/DOX	*ABCB1*	[51]
	Gastric	−/Trastuzumab	*FBXW7*	[230]
- 224	Lung	+/CDDP	*P21(WAF1/CIP1)*	[62]
- 298	Breast	−/DOX	*MDR1*	[184]
- 320	Colon	−/5-FU, L-OHP	*FOXM1*	[231]
- 320a	Breast	−/DOX	*TRPC5, NFATC3*	[33]
- 320c	Pancreatic	+/GEM	*SMARCC1*	[232]
- 328	Bresat	−/MX	*ABCG2*	[185]
- 337-3p	NSCLC	−/PTX	*STAT3, RAP1A*	[233]
- 338-3p	HCC	−/Sorafenib	*HIF-1α*	[234]
- 340	HCC	−/CDDP	*Nrf2*	[45]
- 370	Ovarian	−/CDDP	*ENG*	[84]
- 375	Breast	−/TAM	*MTDH*	[235]
- 449a	Ovarian	−/CDDP	*NOTCH1*	[87]
- 451	Colorectal	−/Irinotecan	*COX-2*	[236]
	Breast	−/PTX	*YWHAZ*	[237]
- 452	Breast	−/DOX	*IGF-1R*	[30]
- 486	CML	+/Imatinib	*FOXO1, PTEN*	[105]
- 487a	Breast	−/MX	*ABCG2*	[186]
- 489	Ovarian	−/CDDP	*Akt3*	[31]
	Breast	−/DOX	*Smad3*	[238]
- 491-3p	HCC	−/multidrug	*ABCB1, SP3*	[239]
- 497	Colorectal	−/5-FU	*Smurf1*	[240]
- 503	Lung	−/CDDP	*BCL-2*	[166]
	Colorectal	+/L-OHP	*PUMA*	[241]
- 513a-3p	Lung	−/CDDP	*GSTP1*	[66]
- 519d	Breast	−/CDDP	*MCL-1*	[242]
- 591	Ovarian	−/PTX	*ZEB1*	[99]
- 587	Colorectal	+/5-FU	*PPP2R1B*	[243]
- 650	Lung	+/Docetaxel	*ING4*	[40]
- 663	Breast	+/Docetaxel, Cyclophosphamide	*HSPG2*	[244]
- 1915	Colorectal	−/L-OHP, DOX	*BCL-2*	[80]
- 3656	Pancreatic	−/GEM	*RHOF*	[245]

+: Promote drug resistance; -: Reduce drug resistance

### Breast cancer

Breast cancer is the most common malignant tumor in women. It accounts for 31% of all female cancers, and the number of new cases reached 1.677 million in 2012 [24]. Some chemotherapy drugs, such as paclitaxel (PTX), 5-fluorouracil (5-FU), and doxorubicin/adriamycin (DOX), are used to treat breast cancer, but most of them may eventually lead to chemoresistance and treatment failure. In MCF-7 breast cancer cell line, both the increased and reduced expression of miRNA-21 affected their susceptibility to DOX. The upregulation of miRNA-21 is accompanied with the downregulation of phosphatase and tensin homolog (PTEN). Overexpression of PTEN could mimic the same effects of miRNA-21 inhibition and decrease the resistance of MCF-7 cells to DOX. This showed that miRNA-21 can promote DOX-resistance by downregulating PTEN in breast cancer cells [25]. The similar effect can be seen for trastuzumab, an antibody commonly used in anti-epidermal growth factor receptor 2 (HER2) treatment in breast cancer. In the breast cancer cell line MDA-MB-453, miRNA-21 also conferred trastuzumab resistance via PTEN silencing. Administering miRNA-21 antisense oligonucleotides could restore trastuzumab sensitivity in drug-resistant breast cancer xenografts by inducing PTEN expression [26]. In contrast to miRNA-21, miRNA-137 could reduce MCF-7 drug resistance to DOX. This effect was achieved by targeting Y-box-binding protein-1 (YB-1) and then downregulation of p-glycoprotein (P-gp) by miRNA-137 [27]. Additionally, miRNA-149 [28], miRNA-195 [29], miRNA-452 [30], miRNA-489 [31], miRNA-181a [32], and miRNA-320a [33] also reduced the sensitivity of breast cancer to DOX, and their various targets are shown in Table 1.

Clinical studies show that more than two thirds of breast cancer patients are estrogen receptor (ER) positive; for these cases, an ER inhibitor, tamoxifen (TAM), is commonly administered with generally good results. However, a considerable part of patients following treatment eventually developed resistance to TAM. Recently, it has been demonstrated that some miRNAs participate in the regulation of this process. By comparing miRNA expression profiles between ERα-positive and ERα-negative breast cancer cell lines and primary tumors, Zhao et al. [34] found that the expression of miRNA-222 and miRNA-221 in ERα-negative cells was elevated. Further research found that miRNA-221 and miRNA-222 directly interact with the 3′-UTR of ERα mRNA. Ectopic expression of miRNA-221 and miRNA-222 in MCF-7 and T47D cells resulted in a decrease in the production of ERα protein, but not in the expression of the mRNA. Notably, miRNA-221- and/or miRNA-222-transfected MCF-7 and T47D cells became resistant to TAM compared with vector-treated cells. Furthermore, knockdown of miRNA-221 and/or miRNA-222 sensitized MDA-MB-468 cells to TAM-induced cell growth arrest and apoptosis. In contrast, the let-7 family could sensitize MCF-7 to TAM by directly targeting ERα-36, a variant of ERα. ERα and ERα-36 had different function in cells since they regulated different downstream signaling pathways [35], which indicated the complexity of ERα in regulating chemoresistance.

Forkhead box group O (FOXO) is a major member of the forkhead transcription factor family, which induces cell apoptosis via pro-apoptotic proteins such as BCL2-like 11 (BIM), p27, and BCL2 interacting protein 3 (BNIP3). MiRNA-155 can directly target FOXO3a and decrease its expression, which results in breast cancer cells acquiring resistance to a variety of chemotherapy drugs, including DOX, etoposide (VP-16), and PTX [36]. In addition, other miRNAs, such as miRNA-203, miRNA-125b, miRNA-34a, and miRNA-663, were found elevated in chemoresistant breast cancer cells, and knockdown them could partially recover the sensitivity of cancer cells to drugs like TAM, docetaxel, 5-FU, and cisplatin (CDDP) [37–40]. These studies indicate the potential of miRNAs in combination with chemotherapy for antitumor treatment.

### Liver cancer

Hepatocellular carcinoma (HCC) was the sixth most common cancer globally, with over 782,000 new cases in 2012 [1]. Because of up to 120 million hepatitis B virus (HBV) carriers, China has a high incidence of liver cancer. The mortality of HCC patients ranks third among all malignant tumors [41]. HCC patients had high recurrence rate after surgery or chemotherapy, and the 5-year survival rate is only about 50% [42]. Thus, there is an urgent need to study the mechanisms of chemoresistance in HCC.

CDDP is a broad-spectrum anticancer drug, which causes DNA adducts and crosslinks, ultimately generating double-strand breaks during replication. Studies have found that changes in the expression of miRNA-96 [43], miRNA-182 [44], miRNA-340 [45], miRNA-130a [46], and miRNA-199a-5p [47] could either increase or reduce the sensitivity of HCC to CDDP.

Sorafenib is a kinase inhibitor that can directly or indirectly inhibit tumor cellular growth; however, in terminal HCC patients, sorafenib drug resistance is common. By evaluating the cytotoxicity of sorafenib in HBV-positive/negative HCC cell lines, Mao et al. [48] found that the half maximal inhibitory concentration (IC50) of sorafenib was significantly higher in HBV-positive HCC cells than in those without HBV infection. Significant downregulation of miRNA-193b was observed in HBV-positive HCC cells and tissues. The activity of the myeloid cell leukemia-1 (*Mcl-1*) 3′-UTR reporter was inhibited by co-transfection with a miRNA-193b mimic, which implies that restoring the expression of miRNA-193b sensitized HBV-associated HCC cells to sorafenib treatment, and facilitated sorafenib-induced apoptosis. Studies suggest that activation of the transforming growth factor-β (TGF-β) and PI3K/Akt signaling pathways in HCC cells resulted in an acquired resistance to sorafenib. MiRNA-216a/217 could promote the activation of the TGF-β pathway by downregulating mothers against decapentaplegic homolog 7 (SMAD7), which implied that miRNA-216a/217 could induce sorafenib resistance in HCC [49].

ATP-binging cassette (ABC) transporter proteins mediated tumor resistance is one of the most classic tumor resistance mechanisms [50]. MiRNA-223 [51] and miRNA-133a [52] could target *ABCB1* and *ABCC1*, respectively, increasing the sensitivity of HCC cells to DOX.

Comparing the expression profiles of miRNAs in 5-FU-resistant cells with those of their parental cell lines by using miRNA microarrays, Shi et al. [53] found an increased expression of miRNA-141 in 5-FU-resistant cells. Further studies have found that miRNA-141 could induce HepG2 resistant to 5-FU. Using a similar method, Ma et al. [54] compared the IC50 of 5-FU in 8 different types of HCC cells and then compared the miRNA expression profiles of 5-FU-sensitive and -resistant cells by using miRNA sequencing. The results showed that miRNA-193a-3p was significantly upregulated in drug-resistant cells. MiRNA-193a-3p could inhibit serine/arginine-rich splicing factor 2 (SRSF2), a protein that upregulates the expression of pro-apoptotic splicing form of caspase 2 (*CASP2L*). Therefore, the increase in miRNA-193a-3p conferred HCC tolerance to 5-FU. In contrast, miRNA-195 [55] and miRNA-125b [38] increased the sensitivity of HCC to 5-FU by downregulating anti-apoptotic proteins BCL2-like 2 (Bcl-w) and Hexokinase II, respectively.

### Lung cancer

According to 2012 data, lung cancer was the first common cancer in the world with 1.825 million new cases and with a high mortality rate [1]. Pathologically, lung cancer can generally be divided into non-small cell lung cancer (NSCLC), which accounts for 80% of the cases, and small cell lung cancer (SCLC), which is more malignant. The total 5-year survival rate of lung cancer is about 15% [56].

CDDP is a first-line chemotherapeutic treatment for lung cancer. Studies showed that let-7c [57], miRNA-31 [58], miRNA-138 [59], miRNA-182 [60], miRNA-205 [61], miRNA-224 [62], miRNA-106a [63], miRNA-15b [64], miRNA-27a [65], miRNA-513a-3p [66], miRNA-34a [67], and miRNA-92b [68] could regulate CDDP tolerance of NSCLC by targeting different genes. ABCC2 is an ATP-dependent transport protein, which increases the efflux of drugs and reduces their intracellular concentration. Over expression of *ABCC2* can induce tumor cells resistance to a series of drugs, including CDDP. BCL2-like 1 (Bcl-xl) is a member of the anti-apoptotic protein family, which help resist apoptosis induced by chemotherapeutics. Let-7c is able to target *ABCC2* and *Bcl-xl* simultaneously, reducing their expression, and promoting sensitivity of A549 cells to CDDP [57]. However, another member of the ABC transport protein family, ABCB9, could be inhibited by miRNA-31, thus improving the resistance of NSCLC cells to CDDP [58]. Similarly, ABCA1 could be inhibited by miRNA-106a to improve the resistance of cells to CDDP as well [63].

Another mechanism of drug resistance is the increase in DNA damage repair. Excision repair cross-complementation group 1 (ERCC1) is a member of DNA excision repair family, and increasing the expression of ERCC1 may increase repair rate of DNA damage, so as to improve cell resistance to DNA alkylating agent CDDP. MiRNA-138 can target and downregulate *ERCC1*, thereby reducing NSCLC cell resistance to CDDP [59].

In addition, some pro-apoptotic proteins, such as PTEN and programmed cell death-4 (PDCD4), can be inhibited by miRNA-92b, miRNA-182, and miRNA-205 and improve the resistance of NSCLC to CDDP [60, 61, 68]. MiRNA-15b and miRNA-27a could target and inhibit phosphatidylethanolamine-binding protein 4 (PEBP4) and raf kinase inhibitory protein (RKIP), respectively, to regulate the process of epithelial-mesenchymal transition (EMT) and participate in regulation of CDDP resistance [64, 65].

Gefitinib is an EGF receptor (EGFR) tyrosine kinase inhibitor (TKI), commonly used in the treatment of advanced or metastatic NSCLC. However, these patients eventually develop resistance to EGFR-TKI. Gao et al. [69] revealed that miRNA-138-5p showed the greatest downregulation in gefitinib-resistant cell model (PC9GR). Re-expression of miRNA-138-5p was sufficient to sensitize PC9GR cells and another gefitinib-resistant NSCLC cell line, H1975, to gefitinib. These data fully demonstrated the diverse functions of miRNA.

Huang et al. [40] found that high miRNA-650 expression was an independent prognostic factor for survival of cancer patients. Additionally, they found that the level of miRNA-650 in lung adenocarcinoma (LAD) tissues was correlated with the response of patients to docetaxel-based chemotherapy. Silencing miRNA-650 could increase the in vitro sensitivity of resistant LAD cells to docetaxel, whereas upregulation of miRNA-650 decreased the sensitivity of parental LAD cells to docetaxel both in vitro and in vivo. Additionally, silencing of miRNA-650 could enhance the caspase-3-dependent apoptosis, which might be correlated with the decreased ratio of Bcl-2/Bax. Feng et al. [70] found that miRNA-200b expression was downregulated in tumor tissues of NSCLC patients after docetaxel treatment compared to that before treatment, and the downregulation of miRNA-200b was positively correlated with high expression of E2F transcription factor 3 (*E2F3*). Ectopic expression of miRNA-200b could reverse the resistance of NSCLC to docetaxel.

MiRNA-200b can also regulate the resistance of SCLC to chemotherapeutic drugs. Studies showed that ectopic expression of miRNA-200b could target zinc finger E-box-binding homeobox 2 (*ZEB2*), and make SCLC sensitive to drugs such as CDDP, VP-16, and adriamycin (ADM). Meanwhile, compared to that in parental sensitive cells, the expression of miRNA-200b was downregulated in resistant cells [71].

### Colorectal cancer

MiRNA-21 has been found to promote chemoresistance in many cancers. In colorectal cancer cells, miRNA-21 can significantly inhibit G2/M cycle arrest and apoptosis induced by 5-FU. In vivo experiments demonstrated that overexpression of miRNA-21 could significantly reduce the therapeutic effect of 5-FU and induce 5-FU resistance in mice [72]. Besides miRNA-21, miRNA-10b [73], miRNA-20a [74], and miRNA-23a [75] could also improve the resistance of colorectal cancer cells to 5-FU. Studies have shown that miRNA-10b, which made the colorectal cancer cells resistant to 5-FU via downregulating pro-apoptotic protein BIM, was an independent prognostic factor for the survival of patients. Chai et al. [74] found that the chemotherapeutic-resistant cell line SW620 exhibited higher miRNA-20a expression than the chemotherapeutic-sensitive cell line SW480. Knockdown of miRNA-20a sensitized SW620 cells to chemotherapeutic agents, whereas overexpression of miRNA-20a in SW480 cells resulted in chemoresistance. MiRNA-20a promoted cellular drug resistance by targeting the B cell lymphoma-2 (Bcl-2) family member *BNIP2*, which plays an important role in the mitochondrial-mediated apoptosis pathway. MiRNA-23a promoted the resistance of cells to 5-FU by targeting pro-apoptotic protein apoptosis-activating factor-1 (*APAF-1*) [75]. Other miRNAs, such as miRNA-96 [76] and miRNA-34a [77], could reduce the resistance of colorectal cancer cells to 5-FU.

Oxaliplatin (L-OHP), another platinum anticancer drug, is often used to treat metastatic colorectal cancer, or as adjuvant treatment after surgical resection of the Dukes C colon cancer. Studies have shown that miRNA-153 [78], miRNA-203 [79], and miRNA-20a [74] could regulate the resistance of colorectal cancer to L-OHP. Zhang et al. [78] found that the expression of miRNA-153 increased with the progression of colorectal cancer. Functional experiments showed that miRNA-153 not only promoted the metastasis of colorectal cancer, but also improved the resistance of cells to L-OHP. Zhou et al. [79] revealed that miRNA-203 was upregulated in three out of three L-OHP-resistant colorectal cancer cell lines by microRNA array screening. Exogenous expression of miRNA-203 in chemo-naïve colorectal cancer cells induced L-OHP resistance, whereas knockdown of miRNA-203 sensitized chemoresistant colorectal cancer cells to L-OHP. Research showed that miRNA-203 targeted ataxia telangiectasia mutated (ATM), which mediated DNA damage response in cells. The anti-apoptotic protein Bcl-2 was also involved in drug resistance of colorectal cancer. The expression of miRNA-1915 decreased in L-OHP-resistant cells compared to that in the parental sensitive cells. MiRNA-1915 played a chemoresistance regulatory role by targeting 3′-UTR of *Bcl-2* mRNA. Therefore, overexpression of miRNA-1915 sensitized the cells to drugs, including L-OHP [80].

### Ovarian cancer

Ovarian cancer is the deadliest cancer of the female reproductive system [81]. For advanced ovarian cancer, the first line of chemotherapy is the combination of CDDP/carboplatin with PTX or other chemotherapy drugs. At present, the response of miRNA regulation in ovarian cancer cells to CDDP is the most studied.

Studies show that miRNAs such as let-7 [82], miRNA-9 [83], miRNA-370 [84], miRNA-489 [31], miRNA-130b [85], miRNA-199b-5p [86], and miRNA-449a [87] could reduce the CDDP resistance of ovarian cancer cells. Their targets including genes related to the regulation of cell cycle, proliferation, and apoptosis, such as enhancer of zeste homolog 2 (*EZH2*), cyclin D1 (*CCND1*), AKT serine/threonine kinase 3 (*Akt3*), angiogenesis-related genes endoglin (*ENG*), transport protein gene *ABCC5*, and the DNA repair gene breast cancer suppressor protein (*BRCA1*). Some other miRNAs, such as miRNA-21 [88] and miRNA-93 [89] could promote ovarian cancer cells resistance to CDDP. Their target genes were the proapoptotic genes *PDCD4* or Bcl-2-antagonist/killer 1 (*Bak1*), and the tumor suppressor gene *PTEN*, respectively.

In addition, miRNA-106a and miRNA-130a not only promoted ovarian cancer cell resistance to CDDP, but also improved the sensitivity of cells to CDDP at the same time. The cause of this contradictory result is the miRNA characteristic of being able to target multiple genes, which, in this case, play different regulatory effects in chemoresistance. For example, miRNA-106a promoted drug resistance via targeting *PDCD4* [90], whereas miRNA-130a promoted drug resistance via targeting *PTEN* [91]. However, miRNA-106a also is directed to anti-apoptosis gene *Mcl-1* [92], and miRNA-130a to anti-apoptosis gene X-linked inhibitor of apoptosis (*XIAP*) [93] to play a chemoresistance inhibition role, examples which vividly descript the complexity of miRNA regulation.

For taxanes, Boyerinas et al. [82] found that let-7 family targets the insulin-like growth factor mRNA-binding protein 1 (IMP-1), which destabilizes the mRNA of multidrug resistance 1 (MDR1), making cells more sensitive to taxol. Introducing let-7 g into DOX-RES cells, which express both IMP-1 and MDR1, reduced the expression of both proteins rendering the cells more sensitive to treatment with either taxol or vinblastine, without affecting the sensitivity of the cells to carboplatin, a non-MDR1 substrate. This indicated that let-7 regulated chemoresistance via targeting *IMP-1* was *MDR1* dependent.

Other miRNAs that regulate resistance of ovarian cancer to taxanes are the miRNA-200 family. Taxanes cause cell cycle arrest and apoptosis by binding to and inhibiting the depolymerization of the β-tubulin subunit of microtubules. Studies showed that miRNA-200 can target this subunit and regulate the resistance of ovarian cancer cells to taxanes. For example, Cochrane et al. [94] found that in ovarian cancer cells, miRNA-200c can not only target and inhibit *ZEB1* and *ZEB2* to repress epithelial to mesenchymal transition, but also inhibit the class III β-tubulin (*TUBB3*) gene. The study demonstrated that restoration of miRNA-200c increases sensitivity to microtubule-targeting agents by 85%. Further experiments [95] showed that introduction of a *TUBB3* expression construct lacking the miRNA-200c target site into cells transfected with miRNA-200c mimic results in no change in sensitivity to PTX. Lastly, the authors also proved that the ability of miRNA-200c to enhance sensitivity to PTX is not due to an increased proliferation rate of cancer cells. Because expression of *TUBB3* is a common mechanism of resistance to microtubule-binding chemotherapeutic agents in many types of solid tumors, the ability of miRNA-200c to restore chemosensitivity to such agents may be explained by its ability to reduce TUBB3. Additionally, Cittelly et al. [96] found that miRNA-200c increases sensitivity to taxanes in vitro by targeting the *TUBB3* gene, and it was downregulated in ovarian cancer cell lines and stage III ovarian tumors, and low levels of miRNA-200c correlates with poor prognosis. Restoration of miRNA-200c in an intraperitoneal xenograft model of human ovarian cancer results in a decreased tumor formation and tumor burden. Furthermore, even in established tumors, restoration of miRNA-200c, alone or in combination with PTX, results in significantly decreased tumor burden. This suggested that restoration of miRNA-200c immediately before cytotoxic chemotherapy may allow for a better response or lower effective dose. Therefore, the combination of miRNA-200c with an anti-proliferating drug could be a better treatment to prevent invasiveness of cancers as well as tumor growth both in primary and in metastatic sites [97].

MiRNA-31 [98] and miRNA-591 [99] are also involved in the resistance of ovarian cancer cells to taxane. Another miRNA, miRNA-130b not only regulates the resistance of cells to CDDP, but also regulates the drug resistance of cells to taxol [85]. MiRNA-130b hypermethylation was found in ovarian cancer tissues as well as in drug-resistant cell lines, and the methylation level was negatively correlated with its expression. Restoration of miR-130b expression sensitized these cells to CDDP and taxol. Colony-stimulating factor 1 (*CSF-1*) was a direct target of miRNA-130b. Knockdown of *CSF-1* sensitized ovarian cancer cells to anticancer drugs and could partially attenuate the resistance inducing effect of miRNA-130b inhibitors.

### Prostate cancer

Prostate cancer is generally androgen-sensitive at the initial diagnosis, and as a result, most of the patients are treated with anti-androgen therapy. However, many patients eventually develop androgen-refractory cancer after 12–18 months of anti-androgen treatment and become resistant to chemotherapy drugs [81].

MiRNA-148a expression level was found lower in PC3 and DU145 hormone-refractory prostate cancer cells in comparison to normal human prostate epithelial cells (PrEC) and hormone-sensitive prostate cancer cells (LNCaP). Transfection with miRNA-148a precursor inhibited cell growth, cell migration, and invasion, as well as increased the sensitivity to the anticancer drug PTX in PC3 cells. The study identified mitogen- and stress-activated protein kinase (*MSK1*) as a direct target of miRNA-148a. In PC3PR cells, a PTX-resistant cell line established from PC3 cells, miRNA-148a inhibited cell growth, cell migration, and invasion and also attenuated the resistance to PTX. Furthermore, *MSK1* knockdown reduced the PTX resistance of PC3PR cells, indicating that miRNA-148a has an important tumor inhibition effect on hormone-refractory and drug-resistant prostate cancer [100]. MiRNA-143 could also inhibit the proliferation and migration of prostate cancer cells, which were mediated by the downregulation of Kirsten rat sarcoma viral oncogene (KRAS). There was an inverse correlation between expressions of miRNA-143 and KRAS protein in prostate cancer samples. Moreover, overexpression of miRNA-143 in prostate cancer cells suppressed their proliferation and migration and increased their sensitivity to docetaxel by targeting the EGFR/RAS/MAPK pathway. This indicated that miRNA-143 could be used alone or combined with docetaxel to inhibit the development of prostate cancer [101].

Previous studies have demonstrated that miRNA-34a could regulate the resistance of prostate cancer cells to PTX [102]. Similar to miRNA-148a expression, miRNA-34a expression is also reduced in PC3PR cells compared to that in PC3 cells, and its target was determined to be sirtuin 1 (*SIRT1*). MiRNA-34a overexpression attenuated PTX resistance of PC3PR cells. Introduction of miRNA-34a precursor into PC3PR cells resulted in a decrease in human antigen R (*HuR*), *Bcl-2*, and *SIRT1* expression, and in inhibition of *SIRT1* 3′-UTR activity. *HuR* knockdown reduced *SIRT1* and *Bcl-2* expression. These results suggested that miRNA-34a directly and/or indirectly regulated *HuR* expression via interacting with the 3′-UTR of *SIRT1* and *Bcl-2* mRNAs, thereby controlling the expression of certain proteins. Thus, in PC3PR cells, reduced expression of miRNA-34a confers PTX resistance via upregulation of *SIRT1* and *Bcl-2* expression. These findings suggested that miRNA-34a could be a promising therapeutic target for drug resistance in hormone-refractory prostate cancer.

### Leukemia

Chronic myeloid leukocyte (CML) is one of the most studied cancers. It is characterized by the philadelphia (Ph) chromosome, i.e., the oncogene *c-abl* on chromosome 9 is translocated to the site of the breakpoint cluster region (*bcr*) gene on chromosome 22, assembling the fusion gene *bcr-abl*. The fusion protein BCR-ABL possesses tyrosine kinase activity. Imatinib is an inhibitor of BCR-ABL tyrosine kinase and has therapeutic effects on Ph-positive CML patients. However, with the wide use of imatinib in hospitals, a considerable portion of the patients developed drug resistance. Several mechanisms, including miRNAs, are involved in imatinib resistance in CML. Hershkovitz-Rokah et al. [103] found that miRNA-30e was expressed at low levels in CML cell lines and patient samples. Further experiments verified that miRNA-30e directly targeted ABL mRNA and reduced the translation of ABL protein. Enforced expression of miRNA-30e in K562 cells suppressed proliferation and induced apoptosis, and sensitized them to imatinib treatment. These findings suggested that miRNA-30e acted as a tumor suppressor by downregulating BCR-ABL expression. Another study showed that miRNA-203 could also increase the sensitivity of CML to imatinib. It was found that the expression of miRNA-203 in the bone marrow of patients with CML was downregulated, and the combination of miRNA-203 and imatinib could effectively promote apoptosis [104]. However, other miRNAs, like miRNA-486 could elevate imatinib resistance of CML cells by targeting *FOXO1* and *PTEN* [105].

As one type of anthracycline anticancer therapeutics, daunorubicin (DNR) is one of the most effective drugs in treating leukemia [106]. MiRNA-21 expression was upregulated in the DNR resistant cell line K562/DNR compared to that in its parental line K562. Stable transfection of miRNA-21 into K562 cells induced drug resistance, whereas suppression of miRNA-21 in K562/DNR led to enhance DNR cytotoxicity.

Zhang et al. [107] found that miRNA-125b was highly expressed in pediatric acute promyelocytic leukemia (APL) compared to that in other subtypes of acute myelogenous leukemia (AML) and was correlated with treatment response. Remarkably, miRNA-125b was also found upregulated in leukemic drug-resistant cells, and transfection of a miRNA-125b duplex into AML cells could increase their resistance to the therapeutic drug DOX.

### Others

In 2012, gastric cancer was the third leading cause of cancer-related deaths, and the number of new cases globally was 952,000, ranking fifth among all cancers [1]. Studies have indicated that miRNA-21 can regulate the sensitivity of HER2-positive gastric cancer cells to trastuzumab [108]. Overexpression of miRNA-21 downregulated *PTEN* expression, and increased protein kinase B (AKT) phosphorylation, and vice versa. Additionally, overexpression of miRNA-21 also decreased sensitivity of GC cells to trastuzumab, significantly inhibiting trastuzumab-induced apoptosis, whereas suppression of miRNA-21 expression restored it. MiRNA-181b expression was reduced in gastric cancer cell line SGC7901/vincristine (VCR), and its target gene was *Bcl-2*. Overexpression of miRNA-181b could decrease the levels of Bcl-2 and render the cell more sensitive to the apoptosis induced by VCR [109]. Chang et al. [110] established a CDDP-resistant cell line, SGC7901/DDP, and found that the expression of miRNA-200c and its target gene ras homolog gene E (*RhoE*) are significantly different between SGC7901 and SGC7901/DDP cells. Transfection of pre-miR-200c reduced *RhoE* expression and improved the sensitivity of gastric cancer to CDDP chemotherapy.

CDDP is also used in the treatment of cervical cancer. Ectopic expression of miRNA-214 reduces cell survival, induces apoptosis, and enhances sensitivity to CDDP through directly inhibiting BCL2-like 2 (*Bcl2l2*) expression in cervical cancer HeLa and C-33A cells. And the expression of miRNA-214 itself is regulated by DNA methylation and histone deacetylation [111]. Autophagy was activated in cancer cells during chemotherapy and often contributes to tumor chemotherapy resistance. Zou et al [112] found that autophagic activity in cancer cells increased after CDDP or Taxol treatment, as indicated by the enhanced expression of beclin1, a key regulator of autophagy. MiRNA-30a, a miRNA that targets beclin 1, was significantly reduced in tumor cells treated with CDDP. Forced expression of miRNA-30a significantly reduced beclin 1 and the autophagic activity of tumor cells induced by CDDP. The blockade of tumor cell autophagic activity by miRNA-30a expression significantly increased tumor cell apoptosis induced by CDDP treatment. These results demonstrated that miRNA-30a can sensitize tumor cells to CDDP via reducing beclin 1-mediated autophagy.

Pancreatic cancer is a highly malignant tumor of the digestive system, and the 5-year survival rate of patients is only 1–4% [113]. MiRNA-100 was found markedly downregulated in both pancreatic cancer cell lines and tumor cells from patients. In cancer cells, transfection of miRNA-100 could inhibit their proliferation and increased sensitivity to CDDP. Overexpressing miRNA-100 led to a significant inhibition on tumor formation in vivo. Further experiments showed that the miRNA-100 could target fibroblast growth factor receptor 3 (*FGFR3*) to achieve the functions of inhibiting cell growth and drug resistance [114].

## The mechanisms of deregulated expression of miRNAs

**Fig. 2:**
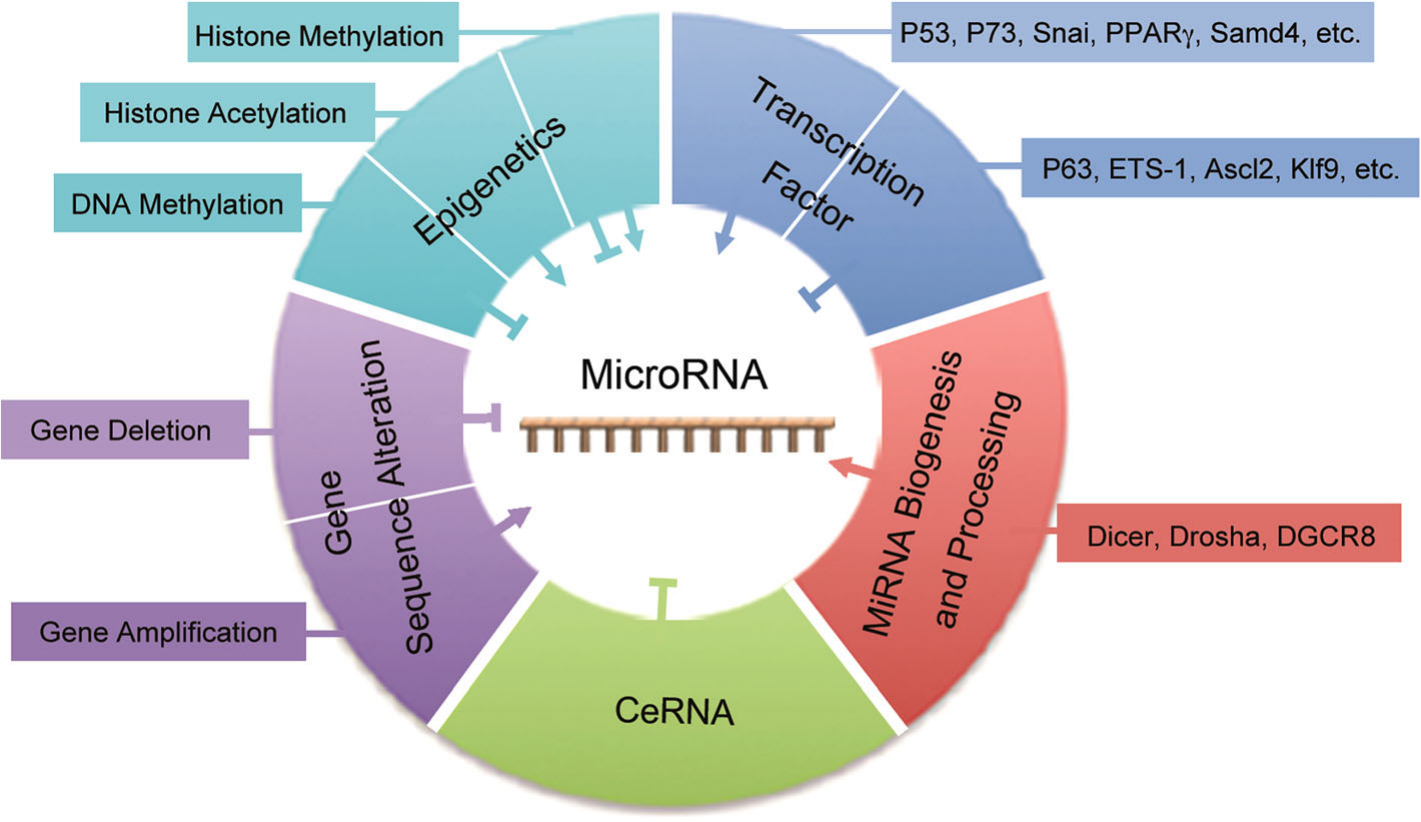
The mechanisms of deregulated expression of microRNAs. Different mechanisms can promote or/and inhibit the expression of miRNA

The abnormal expression of miRNAs could induce malignant transformation of cells, or render tumor cells resistant to chemotherapy drugs. Next, we will review the mechanisms of the dysregulation of miRNA expression in drug-resistant cells. Obviously, studying the mechanisms of miRNA dysregulation will help us overcome the drug resistance. According to results of present studies, the mechanism of miRNA deregulation mainly included the amplification or deletion of miRNA gene, epigenetic regulation, transcription factors deregulation, and the dysregulation of key genes/proteins of miRNA biogenesis and processing [19]. In addition, competitive endogenous RNA (ceRNA) could also reduce the amount of intracellular miRNA, by competitively binding to miRNAs, as illustrated in Fig. 2.

### Gene amplification and deletion

The locations of approximately 50% of all human miRNA genes are at fragile regions or cancer-associated sites, such as minimal region of deletion, amplification, and translocation breakpoints [16]. Genes in these sites were usually more prone to deletion, translocation, or amplification. For example, the deletions at the 13q14 chromosome were most frequently observed in chromosomal abnormalities. Both miRNA-15a and miRNA-16-1 have shown to locate within 13q14 and to be frequently deleted and/or downregulated in more than half of chronic lymphocytic leukemia (CLL) [115]. An opposite example was the miRNA-17~92 family, which is located within the region of 13q31-q32. It was observed that this region is amplified in a variety of tumors, which led to an increase in the amount of mature miRNAs, thus promoting tumor development [116].

Investigations on the relationship between the deregulation of drug resistance associated to miRNAs and gene amplification and deletion are still rare. Studies have indicated that the expression levels of miRNA-219-2 and miRNA-199b were associated with the prognosis and the treatment effect of imatinib of patients with CML. The decrease in miRNA-199b expression could render the CML patients resistant to imatinib [117]. Approximately 15–18% of CML patients showed gene deletions around the translocation breakpoints on der(9)chromosome [118]. Several studies have shown that a deletion in 9q at the same time of the Ph translocation is relevant to the expression of the *bcr-abl* fusion gene. MiRNA-219-2 and miRNA-199b were found to map centromeric to the *abl1* gene within the chromosomal region at 9q34 that was frequently lost in CML patients with der(9) deletions, induced downexpression of miRNA-199b and miRNA-219-2, which resulted in the resistance of CML to imatinib [117, 119].

MiRNA-21 was reportedly upregulated in various cancers, and this could promote drug resistance of tumor cells (Table 1). Hirata et al. [120] proved that overexpression of miRNA-21 in ovarian cancer was caused by the amplification of the chromosomic region 17q23-25, which lead to the low expression of *PTEN* (the target gene of miRNA-21). Chaluvally-Raghavan et al. [121] directly demonstrated the relationship between miRNA gene amplification and tumor drug resistance. They found that miRNA-569, which was overexpressed in a subset of ovarian and breast cancers, at least partly due to 3q26.2 amplification. Downregulation of tumor protein p53 inducible nuclear protein 1 (*TP53INP1*) expression induced by miRNA-569 contributed to the effects of miRNA-569 on cell survival and proliferation. Targeting miRNA-569 sensitizes ovarian and breast cancer cells to CDDP by increasing cell death both in vitro and in vivo. As mentioned before, the upregulation of the miRNA-17~92 family was related to gene amplification, and this resulted in promoting drug resistance in many types of tumors (Table 1). Hayashita et al. [122] found that the miRNA-17~92 cluster, which comprises seven miRNAs and resides in intron 3 of the *C13orf25* gene at 13q31.3, was markedly overexpressed in lung cancers, especially with SCLC cancer histology. The amplification of this region is the direct cause of the overexpression of miRNA-17 family, which enhances the lung cancer cell growth.

### Methylation and histone modifications of miRNA genes

DNA methylation and histone modifications can alter gene expression without altering the DNA sequence. DNA methylation refers to the addition of a methyl group (−CH3) to the cytosine ring of a CpG dinucleotide at the carbon-5 position, which is catalyzed by DNA methyltransferases (DNMTs) [123]. DNA methylation leads to gene silencing and serves as an alternative mechanism of gene inactivation. Post-translational modifications such as acetylation, methylation, and phosphorylation occur on aminoterminal histone tails are strongly associated with active gene transcription or transcriptional repression [124]. Generally, acetylation of histones by histone acetyltransferase (HAT) is associated with gene activation, and hypoacetylation by histone deacetylases (HDACs) is associated with gene inactivation. Histone methylation is associated with both gene activation and silencing. For instance, tri-methylation of lysine 27 of histone H3 (H3K27) is a silencing mark, and methylation of lysine 4 (H3K4) was found at the promoters of active genes [125]. DNA methylation and histone modifications are the epigenetic mechanisms leading to the deregulation of miRNA expression in cancers. There is a collaborative role between DNA methylation and histone modifications in silencing of tumor-suppressive miRNAs in cancer [123].

It was believed that miRNA-34a was a tumor suppressor miRNA; it was downregulated in many kinds of tumor cells and had the effect of improving the sensitivity of tumor cells to chemotherapeutic drugs (Table 1). Lodygin et al. [126] demonstrated that the loss of miRNA-34a expression associated with the methylation of its promoter on CpG island was present in the majority (79.1%) of primary prostate carcinoma samples. Afterward, frequent methylation of the miRNA-34a promoter was found in primary melanoma samples (62.5%), melanoma (43.2%), bladder (33.3%), lung (29.1%), breast (25.0%), kidney (21.4%), pancreatic (15.7%), and colon (13.0%) cancer cell lines. In NSCLC, Gallardo et al. [127] demonstrated that the expression of miRNA-34a was significantly reduced in patients with miRNA-34a hypermethylation compared to that in patients with un-methylated miRNA-34a.

MiRNA-320a was often downexpressed in tumor cells. In chemoresistant cells, downregulation of miR-320a was associated with promoter methylation of the miR-320a coding sequence [33]. Saito et al. [128] found that the combined treatment of human bladder cancer cells with demethylation reagent 5-aza-2′-deoxycytidine (5-Aza-CdR) and the HDAC inhibitor 4-phenylbutyric acid (PBA) has a significant effect on the expression of miRNAs. It could elevate the expression of miRNAs, including miRNA-127. Another example of methylation regulation in drug resistance-associated miRNA is miRNA-149. MiRNA-149 was downregulated and involved in chemoresistance in ADM-resistant human breast cancer cells (MCF-7/ADM). Downregulation of miRNA-149 was related to hypermethylation of its 5′-UTR. With downregulated miRNA-149, the target gene GlcNAc N-deacetylase/N-sulfotransferase-1 (*NDST1*) was upexpressed; thus, the resistance of human breast cancer cells MCF-7 to ADM increased [28]. Yang et al. [85] found miRNA-130b was downregulated in multidrug-resistant ovarian cancer cells. MiRNA-130b hypermethylation was found in ovarian cancer tissues as well as in drug-resistant cell lines, and the methylation level was negatively correlated with its expression. Demethylation with 5-Aza-CdR led to reactivation of miRNA-130b expression in drug-resistant ovarian cancer cell lines, thus increasing the cell sensibility to CDDP and taxol. A more intuitive evidence was that methylation of miR-129-5p CpG island in gastric cancer cells reduced the expression of miR-129-5p and caused the cells to acquire drug resistance. Compared to the expression in parent cells, expression of miR-129-5p was restored in a multidrug-resistant cell line treated with a 5-Aza-CdR [129].

As mentioned above, miRNA-21 is a carcinogenic miRNA in various tissues, and its upregulation can promote tumor development and chemoresistance. Song et al. [130] retrospectively collected 41 cases of advanced pancreatic cancer patients who were sensitive or resistant to gemcitabine (GEM) and assessed levels of serum circulating miRNA-21 to determine a correlation with cytotoxic activity. Histone acetylation in the miRNA-21 promoter was also studied in GEM-sensitive and GEM-resistant pancreatic ductal adenocarcinomas (PDAC) cells. They found that histone acetylation levels at miRNA-21 promoter were increased in PDAC cells after treatment with GEM, and the upregulation of miRNA-21 induced by histone acetylation in the promoter zone was associated with chemoresistance to GEM and enhanced malignant potential in pancreatic cancer cells. Additionally, miRNA-214, which inhibited the development of cervical cancer, was downexpressed by DNA methylation and histone deacetylation, resulting in tumorigenesis and drug resistance [111]. Other examples were miRNA-375 and miRNA-200. Studies showed that the expression of these two miRNAs was downregulated in drug-resistant breast cancer cells. A treatment of a demethylation agent, in combination with a HDAC inhibitor, could restore the expression of these miRNAs in cells [131, 132].

### Abnormality of transcription factors

Transcription factors, also known as trans-acting factors, are proteins that can specifically bind to cis-acting elements in the promoter region of a eukaryotic gene, activating or inhibiting gene transcription, directly or with the help of other proteins.

The most known transcription factor in miRNA regulation is p53. P53 can work as a tumor suppressor, and its expression is upregulated when DNA has been damaged. It is associated with the regulation of hundreds of genes. In 2007, several laboratories almost simultaneously announced that the miRNA-34 family, including miRNA-34a, was a direct target of p53, and could be significantly upregulated by this molecule [133]. MiRNA-34a, which is generally considered to be a tumor suppressor miRNA, improves the sensitivity of tumor cells to chemotherapeutic drugs. P53 directly recognizes the promoters of the miRNA-34 family and activates the transcription of these miRNAs [134]. Two transcription factors associated with p53, p63, and p73 exhibit distinct functions in cells. P73 mediates chemosensitivity, whereas p63 promotes cell proliferation and survival. Ory et al. [135] showed that p63 and p73 could regulate the expression of miRNA-193a-5p. MiRNA-193a-5p, the expression of which was repressed by p63, was activated by pro-apoptotic p73 isoforms in both normal cells and tumor cells in vivo. Chemotherapy caused p63/p73-dependent induction of this miRNA, thereby limiting chemosensitivity due to miRNA-mediated feedback inhibition of p73. Importantly, inhibiting miRNA-193a interrupted this feedback and thereby suppressed tumor cell viability and induced dramatic chemosensitivity both in vitro and in vivo. Another study [136] reported that the p53 family member and p63 isoform, ΔNp63α, promoted miRNA-205 transcription and controlled EMT in human bladder cancer cells.ΔNp63α knockdown reduced the expression of the primary and mature forms of miRNA-205 and of the miRNA-205 “host” gene (*miRNA-205HG*), and decreased the binding of RNA Pol II to the *miRNA-205HG* promoter, inhibiting *miRNA-205HG* transcription.

Another drug resistance-associated miRNA, miRNA-125b, is regulated by multiple transcription factors. In cutaneous T cell lymphoma, overexpression of *c-Myc* repressed miRNA-125b-5p transcription and sensitized lymphoma cells to bortezomib [137]. However, the overexpression of *Snail* dramatically increased the expression of miRNA-125b through the Snail-activated Wnt/β-catenin/TCF4 axis. Snail conferred chemoresistance by repressing Bak1 through upregulation of miRNA-125b. Peroxisome proliferator-activated receptor gamma (PPARγ), a multiple functional transcription factor, has been reported to have antitumor effects through inhibition of proliferation, and induction of differentiation and apoptosis. Luo et al. [138] demonstrated that PPARγ could promote the expression of miR-125b by directly binding to the responsive element in miRNA-125b gene promoter region.

Other chemoresistance-related miRNAs, such as miRNA-320a, miRNA-155, miRNA-200, and miRNA-21, were regulated by transcription factors v-ets erythroblastosis virus E26 oncogene homolog 1 (ETS-1) [33], Smad4 [139], achaete scute-like 2 (Ascl2) [140], and Krüppel-like factor 9 (KLF9) [141], respectively, as shown in Fig. 2.

### Dysregulation of miRNA biogenesis and processing

The process of miRNA biogenesis and maturation is shown in Fig. 1. The whole pathway requires a number of key enzymes and proteins, including two RNA III endonucleases, Drosha and Dicer1, the cofactor DGCR8, the transporter EXP5, cofactor TRBP2, and argonaute (AGO) [142]. During miRNA processing, these proteins are regulated, and deregulation of them may lead to abnormal expression of miRNAs. This article focuses on studies about regulation of the processing of miRNAs which are involved in drug resistance.

Kovalchuk et al. [143], for the first time, showed that DOX-resistant MCF-7 cells (MCF-7/DOX) exhibited a considerable dysregulation of their miRNAome profile and an altered expression of miRNA processing enzymes Dicer and AGO 2. These dysregulated miRNAs include those involved in the regulation of cellular chemoresistance, such as miRNA-451, which targets the *MDR1* gene. Transfection of MCF-7/DOX-resistant cells with microRNA-451 resulted in increased sensitivity of cells to DOX, indicating that correction of altered miRNA expression may have significant implications for therapeutic strategies that aim to overcome cancer cell resistance. Bu et al. [144] directly demonstrated the relationship between Dicer and cellular drug resistance in a different way. They found that in breast cancer cell line MCF-7, knockdown of Dicer by siRNA led to significant G1 arrest and increased sensitivity to CDDP. Moreover, the decreased expression of miRNA-21 accompanied the decrease of Dicer. Another study showed that hyaluronan could mediate the phosphorylation of the stem cell marker, Nanog, in the breast tumor cell line MCF-7. Phosphorylated Nanog then upregulated the RNase III Drosha and the RNA helicase p68. This process led to microRNA-21 production and tumor suppressor proteins (e.g., PDCD4) reduction. All these events contributed to the upregulation of inhibitors of apoptosis proteins (IAPs) and MDR1, resulting in anti-apoptosis and chemotherapy resistance [145]. Kim et al. [146] demonstrated that ribonuclease/angiogenin inhibitor 1 (RNH1) was necessary for pri-miR-21 processing. Further analysis showed that RNH1 directly interacted with the Drosha complex, and PTEN blocked this interaction, decreasing the expression of miRNA-21. That is, PTEN-mediated miRNA-21 expression regulation was achieved by inhibiting the interaction between RNH1 and Drosha.

Pri-miRNAs are cleaved by Microprocessor, which comprises the double-stranded RNase III enzyme Drosha and its essential cofactor, DGCR8, to produce pre-miRNAs [142]. MiRNA-15a/16 was involved in regulation of chemoresistance in tumor cells (Table 1), and its expression was directly correlated to nucleolin expression. Cellular localization was critical for the proper functioning of nucleolin in this pathway; nucleolin directly interacts with DGCR8 and Drosha in the nucleus, thus regulating the expression of miRNA-15a/16 [147]. Interestingly, proteins involved in miRNA processing, such as Dicer, are also regulated by miRNA. It has been shown that imprecise cleavage of a primary or precursor RNA by Drosha or Dicer, respectively, may yield a group of miRNA variants designated as “isomiR.” There are different mechanisms of miRNA-31 regulation in different cells (Table 1) and in its biogenesis, and processing three isoforms (miR-31-H, miR-31-P, and miR-31-M) might be produced, which differ only slightly in their 5′- and/or 3′-end sequences. Chan et al. [148] validated a predicted target gene, *Dicer*, to be a novel target of miR-31, but only miR-31-P could directly repress *Dicer* expression in both MCF-7 breast cancer cells and A549 lung cancer cells, resulting in their enhanced sensitivity to CDDP, a known attribute of Dicer knockdown. This shows that there is a complex relationship between miRNA and its processing proteins.

### CeRNA

The concept of ceRNA was first presented by Salmena et al. in 2011 [149]. The hypothesis suggested that besides mRNA, sequences of pseudogenes, long non-coding ribonucleic acids (lncRNAs), and circular RNAs (circRNAs) contain miRNA response elements (MREs), and they would compete for limited pools of miRNAs as decoys, similar to “miRNA sponges,” thereby reducing the binding of miRNA to its “legitimate” target gene. The ceRNA hypothesis postulated that any RNA transcript that harbors MREs can sequester miRNAs from other targets sharing the same MREs, thereby regulating their function [150]. At present, some studies have indicated that ceRNAs can affect the regulatory effect of chemoresistance-related miRNAs to their target genes [151, 152].

*PTEN* is a tumor suppressor gene, and its expression is downregulated in a variety of tumors. The 3′-UTR of *PTEN* mRNA can be targeted by a variety of miRNAs, thereby inhibiting its expression. *PTENP1* is a pseudogene of the *PTEN* tumor suppression gene (*TSG*), and its mRNA sequence, close to the 3′-UTR, is highly homologous to the same region in *PTEN*. This feature of *PTENP1* shows that it can be used as a ceRNA of *PTEN*. Indeed, the 3′-UTR of *PTENP1* and *PTEN* mRNA both harbor MREs that can bind miRNA-21. Yu et al. [152] found that overexpression of miRNA-21 in clear-cell renal cell carcinoma (ccRCC) might promote cell proliferation, migration, and invasion in vitro, and tumor growth and metastasis in vivo*.* Overexpression of *PTENP1* in cells expressing miRNA-21 reduces cell proliferation, invasion, tumor growth, and metastasis, recapitulating the phenotypes induced by *PTEN* expression. In addition, overexpression of *PTENP1* in ccRCC cells sensitized these cells to CDDP and GEM treatments, in vitro and in vivo. In clinical samples, the expressions of *PTENP1* and *PTEN* were inversely correlated with miRNA-21 expression. CcRCC patients with no *PTENP1* expression have a lower survival rate. These results suggested that *PTENP1* functions as a ceRNA in ccRCC, which suppresses cancer progression.

In short, ceRNA is a relatively new concept. Although there are some studies on the relationship between ceRNAs and tumorigenesis [153, 154], reports on ceRNAs associated with the regulation of chemoresistance is rare. It is believed that more chemoresistance-related ceRNAs will be discovered in the future.

## The mechanisms of miRNAs in regulation of drug resistance

We will recapitulate the know mechanisms of tumor cell resistance to anticancer drugs, as they have been described above. MiRNAs target and regulate mRNAs, including a number of chemoresistance-related mRNAs; that is the fundamental principle behind cancer cell drug-resistance regulation. Integrated current research shows that miRNAs influence cellular drug resistance mainly via regulating cell survival and apoptosis signaling pathways. In addition, miRNAs also can regulate drug targets and the DNA repair system, as well as drug transport and metabolism-related enzymes [7].

### Regulation of cell proliferation, cycle, and apoptosis-related signaling pathways

Quite a large part of anticancer drugs, such as anti-metabolism agents, DNA crosslinking agents, alkylating agents, topoisomerase II inhibitors, and TKIs, act by inhibiting cell proliferation and inducing apoptosis [155]. Drug-resistant cells can tolerate these medications to a certain extent. MiRNAs can regulate drug resistance by regulating key genes in cell proliferation, cell cycle, and apoptosis-related signaling pathways.

*PTEN* can inhibit the PI3K/Akt signaling pathway, thus inhibiting cell proliferation and promoting apoptosis. The inactivation of *PTEN* is closely related to the development of cancer, and a series of miRNAs affect the response of cancer cells to drugs by regulating *PTEN*. For example, miRNA-21 targets *PTEN* in stomach [108] and breast cancer [25, 26], thereby promoting the cell resistance to a variety of drugs. MiRNA-93 targets *PTEN* in breast cancer and upregulates drug resistance of cells to CDDP. In addition, the target genes of miRNA-214 [156], miRNA-486 [105], miRNA-130a [91], miRNA-216a/217 [49], miRNA-92b [68], and miRNA-205 [61] are all *PTEN*, and they regulate cell resistance to a range of drugs in different tumors.

Meanwhile, PTEN is the inhibitor of cyclin-dependent kinase (CDK). MicroRNA-221/222 and miRNA-214 could target *PTEN* to promote the expression of CDK and modify chemoresistance of cells [157, 158].

MiRNAs can play a key role in regulation of drug resistance by targeting key genes of the cell cycle. MiRNAs, such as miRNA-16, miRNA-29, and miRNA-107, can target CDK6 [159–161]. In addition, retinoblastoma (*RB*), *E2F*, and cyclins that initiate S-phase were also targets of some miRNAs. The deregulation of these miRNAs alters the chemoresistance of the cells [162–164].

Bcl-2 is an anti-apoptotic protein, and the *Bcl-2* gene family contains about 30 members, including anti-apoptotic proteins, such as Bcl-2, Bcl-w, Bcl-xl, and Mcl-1, and pro-apoptotic proteins, such as BCL2-associated X (Bax), Bak1, and BCL2-related ovarian killer (Bok). Thus, miRNA-195 [29, 165], miRNA-503 [166], miRNA-1915 [80], miRNA-15a/16 [167], miRNA-181a [32, 168], miRNA-181b [109], miRNA-200b [169], and miRNA-34a [37, 170] could promote sensitivity of cells to anticancer drugs, including CDDP, DOX, TAM, and DNR, through inhibition of Bcl-2. In addition, miRNA-106a [92], miRNA-193b [48], and miRNA-135a/b [171] could target *Mcl-1* to improve CDDP and sorafenib sensitivities in ovarian cancer, lung cancer, and HCC, respectively. Bak1, the pro-apoptotic protein, could be targeted and inhibited by miRNA-125b, thereby enhancing the resistance of APL, ovarian cancer, and breast cancer to CDDP, DOX, and PTX, respectively.

*PDCD4* is also a tumor suppressor gene that can induce apoptosis. Studies showed that miRNA-21, miRNA-106a, and miRNA-182 target *PDCD4*, increasing drug resistance of pancreas cancer, CML, NSCLC, and ovarian cancer to GEM [172] and CDDP [60, 90], respectively.

Hepatocyte growth factor receptor c-Met is one of the hallmarks of multiple tumors, and the mammalian target of rapamycin (mTOR) is involved in PI3K/Akt signaling pathway to promote cell proliferation and survival. Research found that miR-199a-3p could target mTOR and c-Met in HCC cells. Restoring attenuated levels of miR-199a-3p in HCC cells led to G(1)-phase cell cycle arrest, which reduced invasive capability and increased sensitivity to DOX-induced apoptosis [173].

Foxo3a is a transcription factor that can induce cell apoptosis. MiRNA-153 and miRNA-155 could target *Foxo3a* in colorectal and breast cancer cells, respectively, thus increasing resistance to L-OHP, CDDP, DOX, VP-16, and PTX [36, 78].

The role of SIRT1 in tumor is to promote tumor growth by inhibiting the activity of p53. But in the case of a p53 mutation, SIRT1 can also play a role in tumor suppression. In prostate cancer, overexpression of miRNA-34a inhibits *SIRT1*, thereby inducing cell cycle arrest and apoptosis and increasing sensitivity of cells to PTX [102].

P53 ends the cell cycle by blocking CDKs, and the loss of p53 reduces sensitivity of cells to DNA damage and leads to drug resistance. MiRNA-125 could target and inhibit *p53*, thus rendering the cells resistant to DOX [162].

The nuclear factor kappa B (NF-κB) signaling pathway can activate transcription of a series of genes and has the effect of inhibiting apoptosis and promoting tumor cell resistance to chemotherapy. As one of the target genes of miRNA-21, the leucine-rich repeat interacting protein 1 (LRRFIP1) can reduce drug resistance by inhibiting the NF-κB signaling pathway. Therefore, suppression of miRNA-21 by specific antisense oligonucleotides in glioblastoma cell line U373MG led to enhanced cytotoxic effects of teniposide (VM-26) [174].

### Regulation of drug target and drug metabolism-related genes

Anticancer drugs have their suppressive effect from targeting different proteins, and the deregulation of these proteins often renders the drug ineffective, leading to drug resistance. For example, the abnormal expression of *TUBB3* is associated with resistance of ovarian and endometrial cancer to microtubule-targeting agents such as PTX and VCR. Studies showed that ectopic expression of miRNA-200c downregulated TUBB3 and enhanced sensitivity to PTX, VCR, and epothilone B, up to 85% [94]. TAM is an ER antagonist; let-7 and miRNA-221/222 target *ERα-36* and *ERα*, respectively, to reduce or increase the resistance of breast cancer cells to TAM [34, 35]. Vascular endothelial growth factor A (VEGFA) is the target of the angiogenesis inhibitor bevacizumab. MiRNA-126 targets and inhibits *VEGFA*, improving the sensitivity of NSCLC to bevacizumab [175]. These results indicate that the combination of chemotherapy drugs and miRNAs in the treatment of cancer might have a great application value.

Drug-metabolizing enzymes play an important role in cancer treatment. Some drugs need metabolic reactions to convert into an active anticancer form, such as 5-FU, and drugs need to metabolic reactions as well, to convert into inactive products. The most famous drug-metabolizing enzyme group is the cytochrome P450 (CYP) superfamily, which catalyzes the metabolism of the majority of drugs. MiRNA-27b directly targets and downregulates CYP1B1 to affect drug metabolism in breast and cervical cancer [176]. In colon cancer cells, miRNA-148a targets transcription factor pregnane X receptor (*PXR*), which regulates CYP3A4 [177]. Overexpression of miRNA-148a diminished the protein levels of PXR, and this attenuates the induction of CYP3A4. In addition, CYP1A1 is a target of miRNA-892a, and CYP2J2 is negatively regulated by let-7b [178, 179]. It was proved that in transgenic mice, knockdown of CYP3A by miRNA-based shRNA dramatically reduced enzymatic activity [180, 181].

### Regulation of drug transport and DNA damage repair-related genes

The increased expression of drug efflux pump genes is one of the most important mechanisms of drug resistance in tumors [182]. The most common include *ABCC1*, *ABCG2*, and *ABCB1*, which encode the ABC transporter proteins MDR associated protein (MRP1), breast cancer resistance protein (BCRP), and P-gp, respectively. These proteins have similar trans-membrane domains and protect tumor cells by pumping the drugs out of the membrane [183]. In recent years, a large number of studies have found that miRNAs could directly target drug efflux pump, thereby regulating cell resistance to drugs. For example, in HCC, miRNA-223 targeted *ABCB1*, thereby downregulating the cell resistance to DOX [51]. MiRNA-133a targeted *ABCC1*, rendering the cells more sensitive to ADM [52]. MiRNA-298 was found to target *ABCC1* in breast cancer cells, increasing sensitivity of cells to DOX [184]. MiRNA-328 and miRNA-487a could enhance cell sensitivity to mitoxantrone (MX) by targeting *ABCG2* in breast cancer [185, 186]. Other ABC-related genes, such as *ABCA1* and *ABCB9*, were regulated by miRNA-31 and miRNA-106a, and they regulated drug resistance of lung cancer cells to CDDP [58, 63]. Let-7c targeted *ABCC2* in NSCLC, increasing sensitivity to gefitinib [57].

Some chemotherapeutic agents such as CDDP and DOX induce cell apoptosis via DNA damage. Damaged DNA strands require DNA damage repair enzymes to repair the affected sequences. Once the damage DNA chains were repaired, the cell can continue to survive. There are a lot of enzymes involved in the repair of DNA damage, and changes in their expression influence drug resistance to DNA damaging agents. Some miRNAs reverse drug resistance by targeting DNA damage repair-related enzyme genes. For example, miRNA-9 targeted *BRCA1* in ovarian cancer, thereby increasing sensitivity of cells to CDDP [83]. MiRNA-138 targeted *ERCC1* in NSCLC, thus increasing sensitivity to CDDP [59]. Obviously, inhibition of DNA damage repair systems to increase the efficacy of DNA damage agents is a promising approach in the treatment of cancer.

## Conclusions

The discovery of miRNAs has deepened our understanding of human diseases, including cancer. In this article, we have reviewed miRNAs that regulate resistance to chemotherapy in different tumors. The expression of miRNAs is regulated by a series of factors and dysregulated miRNA expression often leads to antitumor drug resistance. The interaction of miRNAs with mRNA, protein, and other non-encoding RNA constitutes their whole regulatory network. The complexity of this network gives miRNAs a wide range of biological functions, which, at the same time, ensure its great potential for clinical application. For example, the “inhibition” or the “replacement” treatment strategy can be performed based on the upregulation or downregulation of miRNAs in cancer cells, respectively [187]. In addition, the expression of miRNAs has been validated as prognosis indicators in patients with certain cancers. Last but not least, miRNAs could act as promising clinical cancer biomarkers [188]. However, there is still a long way before complete clinical applications of miRNAs are fully developed.

As mentioned above, miRNAs are considered to have great potential in the treatment of cancer. Indeed, the efficacy and safety of miRNA-related treatments are better than those of treatments based on siRNA [189]. Although the role of miRNA in reversing drug resistance is unquestionable, there are still several important issues that need to be further addressed. First, due to the heterogeneity of tumor cells and the diversity of anticancer drugs, some miRNAs have different regulatory effects on drug resistance in different types of tumors, some even being the opposite. Therefore, it is necessary to further and extensively confirm the mechanisms and effects of these miRNAs regulating cellular drug resistance and to screen some of the miRNAs with broad-spectrum regulation of resistance for mechanism research and clinical development. Second, in vitro studies are abundant, whereas in vivo studies are still relatively rare. Given the complexity of the animal’s internal environment, some miRNAs that exhibit good regulation of drug resistance in in vitro studies may not necessarily be effective in vivo. Therefore, the effects of most of the miRNAs need to be further verified in vivo. Third, miRNAs are large molecules; therefore, studies regarding their timely and effective targeting and entry into tumor cells in the body require attention. At present, there are very few studies in this field. Methods such as coupling specific tumor ligands onto the surface of the miRNA-based drugs ensure that miRNAs can be transported to tumor tissues to a greater extent, as well as reduce the side effects and improve the safety of miRNA drugs [190]. Fourth, the safety of miRNAs in vivo has yet to be evaluated systematically.

Taking into account that miRNAs can effectively regulate tumor cell resistance to chemotherapy, the use of miRNA in combination with chemotherapy to achieve a better therapeutic effect is promising. For example, Wu et al. [191] tried to combine miRNA-27b with a variety of anticancer drugs. They found that this miRNA could enhance the anticancer effect of chemotherapy by p53 activation and CYP1B1 inhibition, indicating that the miRNA and the drug had obvious synergistic effect in cancer treatment. In addition, some studies have attempted to encapsulate miRNAs with small molecule drugs in a nano-carrier. Some examples include the co-encapsulation of miRNA-205 and GEM in a nano-carrier for pancreatic cancer treatment [192], co-encapsulation of miRNA-34a, and DOX for breast cancer treatment [193]. These studies showed that the combined action of drug-resistance regulatory miRNAs and chemotherapy drugs had a synergistic effect on tumor cells inhibition, which could improve the effects of chemotherapy drugs, and reverse drug resistance to a certain extent. Based on the mechanisms of tumor cell resistance to chemotherapy, the combination of miRNAs with chemotherapy drugs will be a very promising therapeutic regimen for inhibiting or killing tumor cells in the future, which is worth further study.

## Abbreviations

5-Aza-CdR: 5-Aza-2′-deoxycytidine
5-FU: 5-Fluorouracil
ABC: ATP-binging cassette
ADM: Adriamycin
AGO: Argonaute
Akt: Protein kinase B
Akt3: AKT serine/threonine kinase 3
AML: Acute myelogenous leukemia
APAF-1: Apoptosis-activating factor-1
APL: Acute promyelocytic leukemia
Ascl2: Achaete scute-like 2
ATG7: Autophagy-associated gene 7
ATM: Ataxia telangiectasia mutated
AURKA: Aurora kinase A
Bak1: BCL2-antagonist/killer 1
Bax: BCL2 associated X
BCL: B cell lymphoma
BCL2L2/BCL-w: BCL2-like 2
Bcl-xl: BCL2-like 1
Bcr: Breakpoint cluster region
BCRP: Breast cancer resistance protein
BIM: BCL2-like 11
BIRC5: Baculoviral IAP repeat containing 5
BMF: BCL2-modifying factor
Bmi1: B cell-specific Moloney murine leukemia virus insertion site 1
BNIP3: BCL2 interacting protein 3
Bok: BCL2-related ovarian killer
BRCA1: Breast cancer suppressor protein
CASP2L: Pro-apoptotic splicing form of caspase 2
CCND1: Cyclin D1
CcRCC: Clear-cell renal cell carcinoma
CDDP: Cisplatin
CDK: Cyclin-dependent kinase
CeRNA: Competitive endogenous RNA
CHK1: Check point kinase 1
circRNAs: Circular RNAs
CLL: Chronic lymphocytic leukemia
CML: Chronic myeloid leukocyte
COX-2: Cyclooxygenase-2
CSF-1: Colony-stimulating factor 1
CYP: Cytochrome P450
DNMTs: DNA methyltransferases
DNR: Daunorubicin
DOX: Doxorubicin or Adriamycin
E2F3: E2F transcription factor 3
EGF: Epidermal growth factor
EGFR: EGF receptor
EMT: Epithelial-mesenchymal transition
ENG: Endoglin
ER: Estrogen receptor
ERCC1: Excision repair cross-complementation group 1
ETS-1: v-ets Erythroblastosis virus E26 oncogene homolog 1
EZH2: Enhancer of zeste homolog 2
FasL: Fas ligand
FBXW7: F-box and WD repeat domain-containing 7
FGFR3: Fibroblast growth factor receptor 3
FOXM1: Forkhead box M1
FOXO: Forkhead box O
GEM: Gemcitabine
GPR124: G protein-coupled receptor 124
GSTP1: Glutathione S-transferase pi 1
H3K27: Lysine 27 of histone H3
H3K4: Methylation of lysine 4
HAT: Histone acetyltransferase
HBV: Hepatitis B virus
HCC: Hepatocellular carcinoma
HDAC: Histone deacetylase
HER2: Epidermal growth factor receptor 2
HIF-1α: Hypoxia-induced factor 1 alpha
hMSH2: Human DNA MutS homolog 2
HSPG2: Heparin sulfate proteoglycan 2
HuR: Human antigen R
IAPs: Inhibitors of apoptosis proteins
IC50: Half maximal inhibitory concentration
IGF-1: Insulin-like growth factor 1
IGF-1R: Insulin-like growth factor I receptor
IMP: Insulin-like growth factor mRNA-binding protein
ING4: Inhibitor of growth 4
JAG1: Jagged 1
Keap1: Kelch-like ECH-associated protein 1
KLF9: Krüppel-like factor 9
KRAS: Kirsten rat sarcoma viral oncogene
LAD: Lung adenocarcinoma
lncRNAs: Long non-coding ribonucleic acids
L-OHP: Oxaliplatin
LRRFIP1: Leucine-rich repeat interacting protein 1
MAGI2: Membrane-associated guanylate kinase, WW, and PDZ domain-containing protein 2
MAPK: Mitogen-activated protein kinase
Mcl-1: Myeloid cell leukemia-1
MDR1: Multidrug resistance 1
MiRISC: MiRNA-induced silencing complex
miRNA (microRNA): Micro ribonucleic acid
MREs: MiRNA response elements
MRP1: MDR associated protein
MSK1: Mitogen- and stress-activated protein kinase
MTA1: Metastasis-associated protein 1
MTDH: Metadherin
mTOR: Mammalian target of rapamycin
MX: Mitoxantrone
NCOA3: Nuclear receptor coactivator 3
NDST1: N-deacetylase and N-sulfotransferase 1
NFATC3: Nuclear factor of activated T cell isoform c3
NF-κB: Nuclear factor kappa B
NLK: Nemo-like kinase
Nrf2: Nuclear factor erythroid-2-related factor-2
NSCLC: Non-small cell lung cancer
PBA: 4-Phenylbutyric acid
PDAC: Pancreatic ductal adenocarcinomas
PDCD4: Programmed cell death-4
PEBP4: Phosphatidylethanolamine-binding protein 4
P-gp: P-glycoprotein
Ph: Philadelphia
PHIPP2: PH domain and leucine-rich repeat protein phosphatase 2
PI3K: Phosphoinositide 3-kinase
PIK1: Polo-like kinase 1
PKM2: Pyruvate kinase M2
PPARγ: Peroxisome proliferator activated receptor gamma
PPP2R1B: Protein phosphatase 2 scaffold subunit Abeta
pre-miRNA: Precursor miRNA
pri-miRNA: Primary miRNA
PRKCD: Protein kinase C delta
PRKCE: Protein kinase C epsilon
PTEN: Phosphatase and tensin homolog
PTX: Paclitaxel
PUMA: P53 upregulated modulator of apoptosis
PXR: Pregnane X receptor
RAP1A: RAS-related protein 1a
RB: Retinoblastoma
Rho: Ras homolog gene
RISC: RNA-induced silencing complex
RKIP: Raf kinase inhibitory protein
RNA Pol II: RNA polymerase II
RNH1: Ribonuclease/angiogenin inhibitor 1
RUNX3: Runt related transcription factor 3
SCLC: Small cell lung cancer
SIRT1: Sirtuin 1
SLC7A11: Solute carrier family 7 member 11
SMAD7: Mothers against decapentaplegic homolog 7
SMARCC1: SWI/SNF-related, matrix-associated, actin-dependent regulator of chromatin subfamily c member 1
Smurf1: SMAD-specific E3 ubiquitin protein ligase 1
SOCS3: Suppressor of cytokine signaling 3
SRSF2: Serine/arginine-rich splicing factor 2
STAT3: Signal transducer and activator of transcription 3
TAB3: TAK1-binding protein 3
TAK1: TGF-β-activated kinase-1
TAM: Tamoxifen
TGF-β: Transforming growth factor-β
TKI: Tyrosine kinase inhibitor
TMZ: Temozolomide
TP53INP1: Tumor protein p53 inducible nuclear protein 1
TRPC5: Transient receptor potential channel C5
TSG: Tumor suppression gene
TUBB3: Class III β-tubulin
TYMS: Thymidylate synthase
ULK1: Unc-51-like autophagy activating kinase 1
UTR: Untranslated region
VCR: Vincristine
VEGFA: Vascular endothelial growth factor A
VM-26: Teniposide
VP-16: Etoposide
XIAP: X-linked inhibitor of apoptosis
YAP1: Yes-associated protein 1
YB-1: Y-box-binding protein-1
YWHAZ: Tyrosine 3-monooxygenase/tryptophan 5-monooxygenase activation protein zeta
ZEB: Zinc finger E-box-binding homeobox
ZNF217: Zinc finger protein 217
